# Supplementary material for: The community composition variation of Russulaceae associated with the Quercus mongolica forest during the growing season at Wudalianchi City, China
Source: PeerJ. 2020 Feb 12;8:e8527. doi: 10.7717/peerj.8527 (PMC7023826; doi:10.7717/peerj.8527)
Supplement: Supplemental Information 3 [file peerj-08-8527-s003.docx]

>Lactarius mammosus Otu1191

AAGTCGTAACAAGGTTTCCGTAGGTGAACCTGCGGAAGGATCATTATCGTAACAAAATGTGTTGAGGCATGCAACGGCTG

TCGCTGACCAAAAGTCGTGCACGCCAGGGTGTGTCCTCTCACATAAAATCCATCTCACCCTTTTGTGCACCACCGCGTGG

GCACCCTTCGGGGTCGAACAGATCCAGGAGGGGGCTTGCGTTTTCACACAAACCCCTTTTTAAAAGTGTAGAATGACCCC

ACTTTTGCGATAACACGCAATCAATACAACTTTCAACAACGGATCTCTTGGCTCTC

>Lactarius pyrogalus Otu412

AAGTCGTAACAAGGTTTCCGTAGGTGAACCTGCGGAAGGATCATTATCGTACAAAATGTGTGAGGCATGCAAGGGCTGTC

GCTGACTTTTAAACACAAAAGTCGTGCACGCCAGAGTGTGTCCTCTCACATAAAATCCATCTCACCCCTTTGTGCACCAC

CGCGTGGGCACCCTCCGGGATCATACCGATCCAGGAGGGGGCTTGCGTTTTCACACAAAACCCCTTTTTAAAAAGTGTAG

AATGACCTCATTTTTGCGATAACACGCAATCAATACAACTTTCAACAACGGATCTCTTGGCTCTC

>Russula anthracina Otu191

AAGTCGTAACAAGGTTTCCGTAGGTGAACCTGCGGAAGGATCATTATCGTACTACAGAGGTGCTCAGGTTGTCGCTGACC

TTTTTGGTCGTGCACGCCTGAGTGCTCTCAATCCATTTCACCCTTTGTGCATCACCGCGCGGGGTCTCTTCCTCTTGGCT

TGCATCAAGAGGGGAGGTTCGCGTTTTTCATACAAACACCCTTCTAGTTTAGAATGTCATTCATTTGCGATCATACGCAA

TCAATACAACTTTCAACAACGGATCTCTTGGCTCTC

>Russula azurea Otu149

AAGTCGTAACAAGGTTTCCGTAGGTGAACCTGCGGAAGGATCATTATCGTACAACGGAGGTGCGAGGGCTGTCGCTGACTTTCAAAGGTCGTGCACGCCCAAGCGCTCTCTCACACATCCATCTCACCCCCTTTGTGCATCACCGCGTGGGTCCTCCTCACAGAGGGCTCGCGTTTTCACATAAAACTGGATACTGTATAGAATGTTTTCTTTTTTTGTTTGCGGTCACACGCAATCAAT

ACAACTTTCAACAACGGATCTCTTGGCTCTC

>Russula cremeoavellanea Otu138

AAGTCGTAACAAGGTTTCCGTAGGTGAACCTGCGGAAGGATCATTATCGTACAATTGAGGTGCAAGGGCTGTCGCTGACC

TTCAAAGGTTGTGCACGCCCGAGCACTCTTACACATCCATCTTACCCCTTTGTGCATCACCGCGTGGGCCCCCCTTTGCA

GGAGGGCTTGCGTTTTCACATAAAACTTGATACAGTGTAGAGTGGTTTTTTTTTGCGGTCACACGTAATTAATACAACTT

TCAACAACGGATCTCTTGGCTCTC

>Russula cyanoxantha Otu32

AAGTCGTAACAAGGTTTCCGTAGGTGAACCTGCGGAAGGATCATTATCGTATAACGGAGGTGCCGGGGCTGTCGCTAACT

CTGTGCACGCCCTGGTGCTCTCTCACAAAATCCATCTCACCCCCTTGTGCATCACCGCGTGGGTGCTCTCCTTTTGGTTC

GTTCCGAAAGGGGGCGTTTGCGTTTTTATACAAAACCACTGCATGTGTAGAATGTCTTACTATTTGCGATAATGCGCAAT

CAATACAACTTTCAACAACGGATCTCTTGGCTCTC

>Russula exalbicans Otu304

AAGTCGTAACAAGGTTTCCGTAGGTGAACCTGCGGAAGGATCATTATCGTACAACTGAGGCGCGAGGGCTGTCGCTGACT

TTTCGTCGTGCACGCCCGAGCGCTCTCATACATCCACCTCACCCCTATGTGCACCACCGCGTGGGTCCCCTTTCGGGGGG

GCTCGCGTTTTCACACACAAACTCGATACAGTGTAGAATGTCCTTCTTTGCGATCACACGCAATTAATACAACTTTCAAC

AACGGATCTCTTGGCTCTC

>Russula maculata Otu1299

AAGTCGTAACAAGGTTTCCGTAGGTGAACCTGCGGAAGGATCATTATCGTACAACGGAGGTGCAAGGGCTGTCGCTGACC

TTCAAAGGTCGTGCACGCCCGAGCCCTTTCACACCAATCCATCTCACCCTTTTGTGCATCACCGCGTGGGTCCCCCCCTT

TGCCGGGGAGGGCTTGCGTTTTCACATAAAACTTGACACAATGCAGAATGTTTTCTTTTTTTGCGATTATACGCAATCAA

TACAACTTTCAACAACGGATCTCTTGGCTCTC

>Russula olivobrunnea Otu292

AAGTCGTAACAAGGTTTCCGTAGGTGAACCTGCGGAAGGATCATTATTGTACAACGGAGGTGCAAGGGCTGTCGCTGACC

CTCAAAGGTCGTGCACGCCCGAGCGCGCTCTCACACAATCCATCTCACCCCTTTGTGCATCACCGCGTGGGTCCTCCCCC

TTGCGGGAGGGCCTGCGTTTTCACATAAAACTCGATACAGTGTAGAATGTTCATTTTTGCGGTCACACGCAATCAATACA

ACTTTCAACAACGGATCTCTTGGCTCTC

>Russula pallidospora Otu15

AAGTCGTAACAAGGTTTCCGTAGGTGAACCTGCGGAAGGATCATTATCGTACAACGGAGGCGCGAGGGCTGTCGCTGACT

TTTCAAAGGTCGTGCACGCCCAAGTGTTCTCAATCCATCTCACCCCTTGTGCATCACCGCGGGGGCCCCTTTGGGCCTTG

TTCCAAAGGGTGCTCACGTTTTAACACAGACACCCATTTGAATGCAGTGTAGAATGTTCTTCATGTAATGAATCAATAAT

ACAACTTTCAACAACGGATCTCTTGGCTCTC

>Russula pelargonia Otu769

AAGTCGTAACAAGGTTTCCGTAGGTGAACCTGCGGAAGGATCATTATCGTACATCGGGGGTGCGAGGGCTGTCGCTGACC

CTTTAAAAAAGGTCGTGCACGCCCGAGCGCTCTCAAACATCCATCTCACCCCTTTGTGCATCACCGCGTGGGTCCCCTTT

GCGGGGGGCTTGCGTTTTCACATAAAACTCCATACTGTGTAGAATGTTTTCTTTTTGCGCTCACACGCAATCAATACAAC

TTTCAACAACGGATCTCTTGGCTCTC

>Russula puellula Otu114

AAGTCGTAACAAGGTTTCCGTAGGTGAACCTGCGGAAGGATCATTATCGTACAACCGAGGTGCAAGGGCTGTCGCTGACC

CTTCAAAGGTCGTGCACGCCCGAGCGCTCTCACCACAATCCATCTCACCCCTTTTGTGCATCACCGCGTGGGTCCCCCCT

TTGCGGGAGGGCTCGCGTTTTCACATAAAACTTGATACAGTCTAGAATGTTTATTTTTGCGGTCACACGCAATCAATACA

ACTTTCAACAACGGATCTCTTGGCTCTC

>Russula romellii Otu1367

AAGTCGTAACAAGGTTTCCGTAGGTGAACCTGCGGAAGGATCATTATCATACAACAGAGGTGCGAGGGCTGTCGCTGACC

TTTTAAAGGTCGTGCACGCCCGAGTGCTCTCACACATCCATCTCACCCCTTTGTGCACAACCGCGTGAGTTCCCCTGGAA

GGGGGGGCCCACGTTTTTTCACACAAACCTTAAAGCAGTGTAGAATGTATTTTCTTTTTGCGGTGATACGCGATCAATAC

AACTTTCAACAACGGATCTCTTGGCTCTC

>Russula subrubescens Otu157

AAGTCGTAACAAGGTTTCCGTAGGTGAACCTGCGGAAGGATCATTATTGTATAACGGAGGTGCAAGGGCTGTCGCTGACC

TTTAAAGGTCGTGCACGCCTAAGCCCTCTCACACAATCCATCTCACCCCCTTTTGTGCATCACCGCGTGGGTCCTCCCTT

TGCCGGGAGGGCCTGCGTTTTTATATAAAACTTGACACGATGTAGAATGTTTTCTTTTTTGCAATCATATGCAATAAATA

CAACTTTCAACAACGGATCTCTTGGCTCTC

>Russula vitellina Otu129

AAGTCGTAACAAGGTTTCCGTAGGTGAACCTGCGGAAGGATCATTATCGTACAACGGGGGTGCGAGGGCTGTCGCTGACC

TTTCAAAAGGTTGTGCACGCCCGAGCGCTCTCACACATCCATCTCACCCCTTTGTGCATCACCGCGTGGGTCCCCCTTTG

CAGGAGGGCTCACGTTTTCACACAAAACTTGATACAGTGTAGAATGTTATTTTTGCGGTCACACGCAATCAATACAACTT

TCAACAACGGATCTCTTGGCTCTC

>Russula sp 1 Otu83

AAGTCGTAACAAGGTTTCCGTAGGTGAACCTGCGGAAGGATCATTATCGTGTAACCGAGGTGCAAGGGCTGTCGCTGACC

TTTCAAAGGTCGTGCACGCCCAAGCACACTCTCGCACATCCATCTCACCCCTTTTGTGCATCACCGCGTAGGCCCCCCTT

TGCAAGGAGGGTTTGCGTTTTCACATAAAAACTTGATACAGTATATAGAATGTTTCTTTTTTATTTTGCGGTCACACGCA

ATCAATACAACTTTCAACAACGGATCTCTTGGCTCTC

>Russula sp 2 Otu193

AAGTCGTAACAAGGTTTCCGTAGGTGAACCTGCGGAAGGATCATTATCGTACAACAGAGGTGTAAGGGCTGTTGCTGACC

CTTAAAGGTCGTGCACGCCTAAGCCCTCTTACACAATCCATTTCACCCCTTTTGTGCATCACCGCGTGGGTCCTCCCTTT

GCCAGGAGGGCCTGCGTTTTTATATAAAACTTGACACGATGTGGAATGTTTTCTTCTTTTGCGATTATATGCAATCAATA

CAACTTTCAACAACGGATCTCTTGGCTCTC

>Russula sp 3 Otu204

AAGTCGTAACAAGGTTTCCGTAGGTGAACCTGCGGAAGGATCATTATCGTATAACAGAGGTGTAAGGGCTGTCGCTGACC

TTCGAAGGTCGTGCACGCCTAAGCCCTCTCACACAATCCGTCTCACCCCTTTTGTGCATCACCGCGTGGGTCCTCCCTTT

GCCGGGAGGGCCTGCGTTTTTATATAAAAACTTGACATGATGTAGAATGTTTTCTTTTTCTTTTTGCAATTATATGCAAT

CAATACAACTTTCAACAACGGATCTCTTGGCTCTC

>Russula sp 4 Otu37

AAGTCGTAACAAGGTTTCCGTAGGTGAACCTGCGGAAGGATCATTATCGTACAATGGGGGTACGACGGCTGTCGCTGACT

TTAGTCGTGCACGCCCGAGTGCTCTCACATACAAATATCCATCTCACCCCTTTGTGCATCACCGCGTGGGTCCCCCTTCC

TCGGAGGGGGTGCTCACGTTTTTAACATTAAACACCCATTCGAACGTAGTGTAGAATGTTCTTTGCGCGATCACGCGCGA

TCAATACAACTTTCAACAACGGATCTCTTGGCTCTC

>Russula sp 5 Otu128

AAGTCGTAACAAGGTTTCCGTAGGTGAACCTGCGGAAGGATCATTATCGTACAACGGGGGTGCAAAGGCTGTCGCTGACC

TTCCAAAAGGTCGTGCACGCTCGAGCACTCCCATACATCCGTCTCACCTTTGTGCATCACCGCGTGGGTCCCCTTTGCAA

GGAGGGCTCACGTTTTCACACAAAAACTTGATACAGTGTAGAATGTTATTTTTGCGGTCACACGCAATCAATACAACTTT

CAACAACGGATCTCTTGGCTCTC

>Russula sp 6 Otu269

AAGTCGTAACAAGGTTTCCGTAGGTGAACCTGCGGAAGGATCATTATCGTATAACGGGGGTGCAAAGGCTGTCGCTGACC

TTTCAAAAGGTCGTGCACGCTCGAGCACTCTCATACAATCCGTCTCACCTTTGTGCATCACCGCGTGGGTCCTCTTTGCA

GGAGGGCTCACGTTTTCACACAAAAACTCCATACAGTGTAGAATGTTATTTTTGCGGTCACACGCAATCAATACAACTTT

CAACAACGGATCTCTTGGCTCTC

>Russula sp 7 Otu89

AAGTCGTAACAAGGTTTCCGTAGGTGAACCTGCGGAAGGATCATTATCGTACAACGGAGGTGCGAGGGCTGTCGCTGACC

TTTGAAGGTTGTGCACGCCCGAGCCCTCTCGCAATCCATCTCACCCTTTGTGCACCACCGCGTGGGTCCCCCTTTGCGGG

GAGGGCTCGCGTTTTCACATAAAACTCGATACAGTATAGAACGTTTATTTTTGCGGTCACACGCGATCAATACAACTTTC

AACAACGGATCTCTTGGCTCTC

>Russula sp 8 Otu127

AAGTCGTAACAAGGTTTCCGTAGGTGAACCTGCGGAAGGATCATTATCGTGCAACAGAGGCGCGAGGGCTGTCGCCGACT

CTCGTCGTGCACGCCCGAGCGCTCTCACGCATATCCCACCTCACCCCTTTGTGCATCACCGCGTGGGTCGTCCTCACCCT

TTCGGGGGAAAGGGAGGCTCGCGTTTTTTTATACCAAACCCCCTATCGAACGACGTTTTAGAATGTTTTTATGCGATCAA

TACAACTTTCAACAACGGATCTCTTGGCTCTC

>Russula sp 9 Otu282

AAGTCGTAACAAGGTTTCCGTAGGTGAACCTGCGGAAGGATCATTATCGTACAACTGAGGTGCAAGGGCTGTCGCTGACC

TTCAAAGGTCGTGCACGCCCAAGCACTCTTACACAATCCACCTCACCCCTTTGTGCATCACTGCGTGGGCCCCCCTTTGC

AGGAGGGCCTGCGTTTTCACATAAAACTTGATATAGTGTAGAATGGTTTCTTTTGCGGTCACACGCAATTAATACAACTT

TCAACAACGGATCTCTTGGCTCTC

>Russula sp 10 Otu312

AAGTCGTAACAAGGTTTCCGTAGGTGAACCTGCGGAAGGATCATTATCGTACGATGGGGGTACGACGGCTGTCGCTGACT

TGCATAAAAGGTCGTGCACGCCCGAGTGCTCTCACATACAAATATCCATCTCACCCCTTTGTGCATCACCGCGTGGGTCC

CCCCTTCCTCGGAGGGGGGTGCTCACGTTTTTAACATTAAACACCCATTCGAACGTATCGTAGAATGTTCTTTGCGCGAT

GACGCGCGATCAATACAACTTTCAACAACGGATCTCTTGGCTCTC

>Russula sp 11 Otu915

AAGTCGTAACAAGGTTTCCGTAGGTGAACCTGCGGAAGGATCATTATAGTACAACAGAGGTGCCTGGGCTGTCGCCGTCC

TTTAAGGACGTGCACGCCCGGAGTGCTCTCTCACATCCATCTCTCACCCCTTTGTGCATCGCCGCGTGGGGCCCCCTCTT

TTGGCTTGTTCCGGAGGGGGGTTCACGTTTTTACACGAACAACCCATTAATGCATGTGTAGAATGTCTTACTTATTTTAA

ATACAACTTTCAACAACGGATCTCTTGGCTCTC

>Russula sp 12 Otu1075

AAGTCGTAACAAGGTTTCCGTAGGTGAACCTGCGGAAGGATCATTATAGTACATCGGAGGCACCTGGGTTGTCGCTGACC

TTTAAGGACGTGCACGCCCAGAGTGTTCTCTCACATCCATCTCTCACCCCTTTGTGCATAACCGCGTGGGACCCTCTCTT

TTGGCTTGTTCCGGAGGAGGGGTTCACGTTTTTACATGAACAACCCATTAATGCATGTATAGAATGTCCTACTTATTTTT

AAATACAACTTTCAACAACGGATCTCTTGGCTCTC

>Russula sp 13 Otu1808

AAGTCGTAACAAGGTTTCCGTAGGTGAACCTGCGGAAGGATCATTATAGTACAATAGAGGCACCTGGGCTGTCGCTGTCC

TTAAAAGGATGTGCACGCCCAGAGTGCTCTCTCACATCCATCTCACCCCTTTGTGCATCACCGCGTGGGGCCCTCTCTTT

TGGCTTGTTCCAGAGGGAGGGGTTCACGTTTTTACAAAAACAACCCATTAATGCATGTAGTAGAATGTCTTACTTTTTTG

CGGTCACACGCAATCAATACAACTTTCAACAACGGATCTCTTGGCTCTC

>Russula sp 14 Otu1403

AAGTCGTAACAAGGTTTCCGTAGGTGAACCTGCGGAAGGATCATTATCGTACGATGGGGGTACGACGGCTGTCGCTGACT

TTTAGTCGTGCACGCTCGAGTGCTCTCGCATACAAATATCCATCTCACCCCTTTTGTGCATCACCGCGTGGGGTCCCCCC

TTCCTCGGAGGGGGGGGGTGCTCACGTTTTTAAACATTAAATACCCATCCGAACGTTAATGTAGAATGTTCTTTGCGCGA

TGATGCGCGATCAATACAACTTTCAACAACGGATCTCTTGGCTCTC

>Russula sp 15 Otu1647

AAGTCGTAACAAGGTTTCCGTAGGTGAACCTGCGGAAGGATCATTATCGTACAACCGAGGCGCAAGGGCTGTCGCTGACCTTTAAAAGGGTTGTGCACGCTCGAGCGCTCTCACACAATCCATCTCACCCTTTTGTGCATCACCGCGTGGGTCCCCTTTGCGGGGGGCTTGCGTTTTCACATAAAACTCCATACTGTGTAGAATGTTTTCTTTTTGCGCTCACACGCAATCAATACAACTTTCAACAACGGATCTCTTGGCTCTC

>Russula sp 16 Otu1717

AAGTCGTAACAAGGTTTCCGTAGGTGAACCTGCGGAAGGATCATTATCGTACAATGGGGGTACGATGGCTGTCTCGTGCA

CGCCCGAGTGCTCTCACATACAAATATCCATCTCTCACCCCTTTGTGCATCACCGCGTGGGTCCCCTTCCTCGGAGGGGG

TGCTCACGTTTTTAACATCGAACACCCATTCGAACGTAGTGTAGAATGTTCTTTGCGCGACGACGCGCGATCAATACAAC

TTTCAACAACGGATCTCTTGGCTCTC

>L. sp. Otu799

AAGTCGTAACAAGGTTTCCGTAGGTGAACCTGCGGAAGGATCATTATCGTACAAAATGTGTGAGGCATGTGAGGGGCTGT

CGCTGACTTTTAACACAAAAGTCGTGCACGCCTGAGCGTGTCCTCCCGCATAAAATCCATCTCACCCTTTTGTGCACCAC

CGCGTGGGCACCCTTCGGGATTGAACCAATCCGGGAGGGGGCTTGCGTTTTCACACAAACCCCTTTTAAAAAGTGTAGAA

TGACCCTCATTTTTGCGATAACACGCAATCAATACAACTTTCAACAACGGATCTCTTGGCTCTC
