# Supplementary material for: The community composition variation of Russulaceae associated with the Quercus mongolica forest during the growing season at Wudalianchi City, China
Source: PeerJ. 2020 Feb 12;8:e8527. doi: 10.7717/peerj.8527 (PMC7023826; doi:10.7717/peerj.8527)
Supplement: Supplemental Information 4 [file peerj-08-8527-s004.docx]

>Lactarius evosmus_MN737756

TAGGTGACCTGCGGAAGGTCATTATCGTACCAAATGTGTGAGGCATGCAAGGGCTGTCGCTGACTTTTGAACACAAAAGTCGTGCACGTCTGAGTGTGTCCTCTCACATAAAATCCATCTCACCCTTTTGTGCACCACCGCGTGGGCACCCTTCGGGATCAAAAAGATCCAGGAGGGGGCTTGCGTTTTCACACAAACCCCTTTTTAAAAGTGTAGAATGACCCCATTTTTGCGATAACACGCAATCAATACAACTTTCAACAACGGATCTCTTGGCTCTCGCATCGATGAAGAACGCAGCGAAATGCGATACGTAATGTGAATTGCAGAATTCAGTGAATCATCGAATCTTTGAACGCACCTTGCGCCCCTTGGTATTCCGAGGGGCACACCCGTTTGAGTGTCGTGAAATTCTCAACCTTCTCGGCTCCTTCTGGATGCCGAAGGAGGCTTGGACTTTGGAGGCCTTTGCTGGAACCTTTCTCTCTTGAAAGCCAGCTCCTCTTAAATGAATTAGCGGGGTCCTCTTTGCTGATCCTCGACATGTGATAAGATGTTTCCATGTCTTGGTTTCTGGCTCTGTTGCTTTTGGGACCTGCTTCTAACCGTCTCAGACTTGCATCGAGACAATGTTTGAGCGTGTCTCCCTCCTCGGGAAACTCTCTCAACACCATGAACCCTTGACCTCAAATCGGGTGAGACTACCCGCTGAACTTAAGCATATCAATAAGCGGAGGAAAAGAAACTA

>Lactarius torminosus_MN704764

GCTCGTAGGTGACCTGCGGAGGATCATTATCGTACAAAATGTGTGAGGCGTGCAAGGGCTGTCGCTGACTTTTAACGCAAAAGTCGTGCACGCCGGAGCGTGTCCTCTCACATAAAATCCATCTCACCCTTTTGTGCACCACCGCGTGGGCACCCTTCGGAATCAACCCGATCCCGGAGGGGGCTTGCGTTTTCACACAAACCCCTTCTTAAAAGTGTAGAATGTCCCCATTTTTGCGATAACACGCAATCAATACAACTTTCAACAACGGATCTCTTGGCTCTCGCATCGATGAAGAACGCAGCGAAATGCGATACGTAATGTGAATTGCAGAATTCAGTGAATCATCGAATCTTTGAACGCACCTTGCGCCCCTTGGTATTCCGAGGGGCACACCCGTTTGAGTGTCGTGAAAATCTCAACCTTCTCGGTTTCTTCTGGACACCGAAGGAGGCTTGGACTTTGGAGGCCTTTGCTGGCGTCTCTCTCTCTTGAGAGAGCCAGCTCCTCTTAAATGAATTAGCGGGGTCCTCTTTGCTGGTCCTCGACATGTGATAAGATGTTTCCATGTCTTGGTTTCTGGCTCTGTTGCCTTTGGGACCCGCTTCTAACCGTCTCAACGAGACAACGTTTGAGCGCGTCTCCCTTCTCGGGAGACCCTCTCAACCTCACGAACCCTTGACCTCAAATCGGGTGAGACTACCCGCTGAACTTAAGCATATCAATAAGCGGAGGAATCGTCGACCTGCAGCATGCAAGCTTGGCACTGGCCGTCGTTTTACAACGTCGTGACTGGGAAAACCCTGGCGTTACCCAACTTAATCGCCTTGCAGCACATCCCCCTTTCGCCAGCTGGCGTAATAGCGAAGAGGCCCGCACCGATCGCCCTTCCCAACAGTTGCGCAGCCTGAATGGCGAATGGCGCCTGATGCGGTATTTTCTCCTTACGCATCTGTGCGGTATTTCACACCGCAT

>Lactarius trivialis_MN704807

GGTTAGATCGTAGGTGACCTGCGGAGGATCATTATCGTACAAAATGTGTGAGGCATGCTAGGGCTGTCGCTGACAAAAGTCGTGCACGCCAGAGTGTCCTCTCACATTAAATCCATCTCACCCTTTTGTGCACCACCGCGTGGGCACCCTTCGGGATCGAACCGGTCCAGGAGGGGGCTTGCGTTTTCACACAAACCCTCATTTAAAAGTGTAGAATGACCCCATTTTTGCGATAACACGCAATCAATACAACTTTCAACAACGGATCTCTTGGCTCTCGCATCGATGAAGAACGCAGCGAAATGCGATACGTAATGTGAATTGCAGAATTCAGTGAATCATCGAATCTTTGAACGCACCTTGCGCCCCTTGGTATTCCGAGGGGCACACCCGTTTGAGTGTCGTGAATATCTCAACCTTCTCGGTTTCTTCTGGATGCCGAAGGAGGCTTGGACTTTGGAGGCCTCTGCTGGCATCTTTTGAAGGCCAGCTCCTCTCAAATGAATTAGCGGGGTCCTCTTTGCCAACCCTCGACATGTGATAAGATGTTTCCATGTCTTGGTTTATGGCTCTGTTGCTTTTGGGACCCGCTTCTAATCGTCTCAATGACAACGTTTGAGAGCGTCTCCCTTCTCGGGAAACCCTCTCGAACCCACGAACCCTTGACCTCAAATCGGGTGAGACTACCCGCTGAACTTAAGCATAT

>Lactarius vietus_MN704811

ATTCGTATGTGACCTGCGGAAGGTCATTATCGTACAAAATGTGTGAGGCATGCAAGGGCTGTCGCTGACTTTTTAATCACAAAAAGTCGTGCACGCCGGAGCGTGTCCTCTCGCATAAAATCCATCTCACCCTTTTGTGCACCACCGCGTGGGCACCCTTTGGGGTCGAACCGATCCAGGAGGGCGCTTGCGTTTTCACACAAACCCCCTTATAAAAGTGTAGAATGACCCCATTTTTGCGATAACACGCAATCAATACAACTTTCAACAACGGATCTCTTGGCTCTCGCATCGATGAAGAACGCAGCGAAATGCGATACGTAATGTGAATTGCAGAATTCAGTGAATCATCGAATCTTTGAACGCACCTTGCGCCCCTTGGTATTCCGAGGGGCACACCCGTTTGAGTGTCGTGAAAATCTCAACCTCCTCGGTTTCTTCTGGACGCCGAAGGAGGCTTGGACTTTGGAGGCCTTTGCTGGCGTCTCTCGCCAGCTCCTCTCAAATGAATTAGCGGGGTCCTCTTTGCCGATCCTCGACGTGTGATAAGATGTTTCCATGTCTTGGTTTCTGGCTCTGTCGCCTTTGGGACCCGCTTCTAACCGTCTCAAGTCGAGACAACGTTTGAGCGTGTCTCCCTTCTCGGGAAACACCCTCGAGCCCACGAACCCTTGACCTCAAATCGGGTGAGACTACCCGCTGAACTTAAGCATATCAATAAGCGGAGGAAAAGAAACTAACAAGGATTCCCCTAGTAACTGCGAGTGAAGCGGGAAAAGCTCAAATTTAAAATCTGGCGGTCTTTGGCC

>Lactifluus Bertillonii_MN704762

TTCGTAGGTGACCTGCGGAAGGATCATTATCGTACAACGAGAGGTGCCAGGGCTGTCGCTGACCCCCTCTTTTTGAGGTCGTGCACGCCTTGAGTGTCCTTTCAAACATCCACCACACACACCCCTTTTGTGCATCACCGCGTGGGCTCCTTTGGGTCGGTTCGATCCAAGTGGGGCTTGCGCTTTTTACACAAACACCCTTCGAATGCAGTGTAGAATGTCCTTTGCGATCGTATGCGATCAATACAACTTTCAACAACGGATCTCTTGGCTCTCGCATCGATGAAGAACGCAGCGAAATGCGATACGTAATGTGAATTGCAGAATTCAGTGAATCATCGAATCTTTGAACGCACCTTGCGCCCCTTGGCATTCCGAGGGGCACACCCGTTTGAGTGTCGTGAATTCCTCAACCTTCTGCATTTCTTGATGGCAGAAGGCTTGGACTTTGGAGGTTCTTGCTGGCCTCTCCTTCTAGAAGCCAGCTCCTCTTAAATGGATTAGCGGGGTCTGCCTTTAGTCGATCCTTGACGTGATAAGCCATTTCTACGTCTTGGATTCGACTCTTTTGAGACCTGCTTCTAATCGTCTTAATGGGGTGCACTTTTGGACCCACGAACCATTTGACCTCAAATCGGGTGAGACTACCCGCTGAACTTAAGCATATCAATAAGCGGAGGAAATCGTCGACCTGCAGGCATGCAAGCTTGGCACTGGCCGTCGTTTTACAACGTCGTGACTGGGAAAACCCTGGCGTTACCCAACTTAATCGCCTTGCAGCACATCCCCCTTTCGCCAGCTGGCGTAATAGCGAAGAGGCCCGCACCGATCGCCCTTCCCAACAGTTGCGCAGCCTGAATGGCGAATGGCGCCTGATGCGGTATTTTCTCCTTACGCATCTGTGCGGTATTTCACACCGCATATGGTGCACTCTCAGT

>Russula aurata_MN704814

GGCTTCTCTATTACACTCGGATGGCCAAAGACCACCAGATTTTAAATTTGAGCTTTTCCCGCTTCACTCGCAGTTACTAGGGGAATCCTTGTTAGTTTCTTTTCCTCCGCTTATTGATATGCTTAAGTTCAGCGGGTAGTCTCACCCGATTTGAGGTCAAGGTTTTTCTTGTGGGACCAGCCTCGCGCCAACATAGGCAGCGGTGACCAGAAACACCACCATTGTCCGTAAGACGGTTAGGAGCGGGTGTCTAAAGGGACAGTGCCAAAATCCAAAACGTAGAATCACCTTATCACGTCAAGGATCAGCAAAGCAAACCCCGCTAATTCATTTAAGAGGAGCTCTCTTTCAAAAAAGGTGAGAGTGAGCATTGATCCTCCAAGTCCAAAATCCTTTCAAAGAAAAGGATTTTGAGAAATTCACGACACTCAAACGGGTGTGCCCCTCGGAATGCCAAGGGGCGCAAGGTGCGTTCAAAGATTCGATGATTCACTGAATTCTGCAATTCACATTACGTATCGCATTTCGCTGCGTTCTTCATCGATGCGAGAGCCAAGAGATCCGTTGTTGAAAGTTGTATTGATTGCGTATCATCGCAAAAAAAAAAAAAAAAAA

>Russula atroglauca_MN704761

TGACCTGCGGAAGGTCATTATAGTACAACGGAGGCACCTGGGCTGTCGCTGACCTTAAAGGACGTGCACGCCCGGAGTGCTCTCTCACATCCATCTCACCCCTTTGTGCATCACCGCGTGGGGCCCTCTCCTTTGGCTTGTTCCGGGGAGGGGGTTTCACGTTTTTACACGAACAACCCATTAATGCATGTGTAGAATGTCTTACTTATTTTAAATACAACTTTCAACAACGGATCTCTTGGCTCTCGCATCGATGAAGAACGCAGCGAAATGCGATACGTAATGTGAATTGCAGAATTCAGTGAATCATCGAATCTTTGAACGCACCTTGCGCCCCTTGGTATTCCGAGGGGTGCACCTGTTTGAGTGTCGTGAACACCCTCAACCTTCTTGGTTTATCGACCGGGAAGGATTGGACTTTGGAGGTTTTTGTTGCTGGCCTCGTTTGAAGCCAGCTCCTCCTAAATGAATTAGTGGGGTCCGCCGTGCCGATCCTCGACGTGATAAGTACGCTTCTACGTCTTGGGGTTTCGCACCGTTCTCGCTTCCAACTTCGAGTTGTGACTCGACCTTATAAACCTTGACCTCAAATCGGGTGAGACTACCCGCTGAACTTAAGCATATCAATAAGCGGAGGATCTCTAGAGGATCCCCGGGTACCGAGCTCGAATTCGTAATCATGGTCATAGCTGTTTCCTGTGTGAAATTGTTATCCGCTCACAATTCCACACAACATACGAGCCGGAAGCATAAAGTGTAAAGCCTGGGGTGCCTAATGAGTGAGCTAACTCACATTAATTGCGTTGCGCTCACTGCCCGCTTTCCAGTCGGGAAACCTGTCGTGCCAGCTGCATTAATGAATCGGCCAACG

>Russula odorata_MN704822

GTAGGTGACCTGCGGAGGATCATTATCGTACCACCGAGGTGCAAGGGCTGTCGCTGACCTTTGAAGGTCGTGCACGCCCGAGCGCTCTCGCCACAATCCATCTCACCCCTTTGTGCATCACCGCGTGGGTCCCCCTTTGCGGGGAGGGCTCGCGTTTTCACACAAAACTTGATACAGTCTAGAATGTTTATTTTTGCGGTAACACGCAATCAATACAACTTTCAACAACGGATCTCTTGGCTCTCGCATCGATGAAGAACGCAGCGAAATGCGATACGTAATGTGAATTGCAGAATTCAGTGAATCATCGAATCTTTGAACGCACCTTGCGCCCCTTGGCATTCCGAGGGGCACACCCGTTTGAGTGTCGTGAAATTCTCAAAATCCTTTTCTTTGATCGGAATAGGACTTTTGGACTTGGAGGTTCAATGCTCGCTTTCGCCTTTGAAAGCGAGCTCCTCTCAAATGAATCAGTGGGGTCCGCTTTGCTGGTCCTTGACGTGATAAGATGCTTCTACGTTTTGGATTTGGCATTGTTTTCTCCGCTCCTAACTGTCCCCATGGGACAACGATGGTGCTTCGGTCGTCGCCATCTATGGCGGGAGGCTGAACCCACAAAGAACCTTGACCTCAAATCGGGTGAGACTACCCGCTGAACTTAAGCATATCAATAAGCGGAGGAAAAGAAACTAACAAGGATTCCCCTAGTAACTGCGAGTGAAGCGGGAAAAGCTCAAATTTAAAATCTGGT

>Russula foetens_MN704817

AGGTGACCTGCGGAAGGTCATTATCATACAATGGAGGTGCTGGGGTTGTCGCTGACCTTTGAAAGGGTCGTGCACACCTTGGTGCTCTCACATATAATCCATCTCACCCCTTTTGTGCATCACCGCGTGGGGACCCCTTTTGGCTAGTTCTGAGGGGGGTCTTCACGTTTTTACACAGACACCCTTTTAATGCAATGTGTAGAATGTCTTACTTTTTGCGATCATACGCAATCAATACAACTTTCAACAACGGATCTCTTGGCTCTCGCATCGATGAAGAACGCAGCGAAATGCGATACGTAATGTGAATTGCAGAATTCAGTGAATCATCGAATCTTTGAACGCACCTTGCGCCCTTTGGCATTCCGAGGGGCACACCCGTTTGAGTGTCGTGACATTCTCAAAACCTTCTTGGTTTCTTGACCGGGAAGGCTTTGGACTTTGGAGGCTTTTGCTGGCCTTCCTTTGTTGAAGCCAGCTCCTCTGAAATGAATTAGTGGGGTCTGCTTTGCCTATCCTCGACGTGATAAGATGTTTTCTACGTCTTGGGTTTTGCACTGTTCCTGCTTCTAACCGTCTCATAGAAGACAATGGTCAAGTGATTGCCACTTGACCCACAAACCTTGACCTCAAATCGGGTGAGACTACCCGCTGAACTTAAGCATATCAATAAGCGGAGGAAAAGAAACTAACAAGGATTCCCCTAGTAACTGCGAGTGAAGCGGGAAA

>Russula font-queri_MN704820

AGTCTTCGTAGGTGACCTGCGGAAGGATCATTATCGTACAACGGAGGCGCGAGGGCTGTCGCTGACCTTCGAAGGTCGTGCACGTCCGAGCCCTCTTACAATCCATCTCACCCTTTGTGCATCGCCGCGTGGGTCCCCCTTGCGGGAGGGCTCGCGTTTTCACATAAAAACTCTATACAGTGTAGAATGTTTGCTTTTGCGGTCACACGCAATCAATACAACTTTCAACAACGGATCTCTTGGCTCTCGCATCGATGAAGAACGCAGCGAAATGCGATACGTAATGTGAATTGCAGAATTCAGTGAATCATCGAATCTTTGAACGCACCTTGCGCCCCTTGGCATTCCGAGGGGCACACCCGTTTGAGTGTCGTGAAACCCTCAAAAACCCTCTTCTTTGAGGATTTTTGGACTTGGAGGTTCAATGCTCGCTTTTGCATTCGAAAGCGAGCTCCTCTCAAAAGAATCAGTGGGGTCTGCTTTGCTTGTCCTTGACGTGATAAGATGTTTCTACGTTTTGGATTTGACATGGTCCCTGCTCCTAATCGTCTCACGGACAATGATGGTGCTTCGGTCGCCGCCGCCTACCTTGGCGGGGGAGGCTGAGCCCACAAAAATCTTGACCTCAAATCGGGTGAGACTACCCGCTGAACTTAAG

>Russula globispora_MN704821

CCTGCGGAGGATCATTATCGTATAACAGAGGTGTAAGGGCTGTCGCTGACCTTTAAAGGTTGTGCACGCCTAAGCCCTCTCACAATCCATCTCACCCCTTTTGTGCATCACCGCGTGGGTCCTCCCTTTGCCGGGAGGGCCTGCGTTTTTATATAAAACTTGACACAATGTAGAATGTTTTCTTTTTTGCAATTATATGCAATCAATACAACTTTCAACAACGGATCTCTTGGCTCTCGCATCGATGAAGAACGCAGCGAAATGCGATACGTAATGTGAATTGCAGAATTCAGTGAATCATCGAATCTTTGAACGCACCTTGCGCCCCTTGGCATTCCGAGGGGCACACCCGTTTGAGTGTCGTGAAATTCTCAAAACCCCCTTTTCTTTGATCCTTTTTTGGGGTTTGGAAAAGGAGTTTTGGACTTGGAGGTTCAATGCTTGCCTTTCACTTTTGAAAGCGAGCTCCTCTCAAATAAATTAGTGGGGTCCGCTTTGCTGATCCTTAACGTGATAAGATGTTTCTACGTTTTGGATTTGGCACTGTCCTTTGGATGCCTGCTTCTAACTGTCTCGCGGACAATGATGGTGCTCCGGTCACTACCATTTATATTGGTGGAAGGCTGGACCCACAAAAATAAAACCTTGACCTCAAATCGGGTGAGACTACCCGCTGAACTTAAGCATATCAATAAGCGGAGGAAAAG

>Russula acrifolia_MN704839

TAGGTGACCTGCGGAGGATCATTATCGTACTACAGAGGTGCTCAGGTTGTCGCTGACCTTTTTGGTCGTGCACGCCTGAGTGCTCTCAATCCATTTCACCCTTTGTGCATCACCGCGCGGGGTCTCTTCCTCTTGGCTTGCATCAAGAGGGGAGGTTCGCGTTTTTCATACAAACACCCTTCTAGTTTAGAATGTCATTCATTTGCGATCATACGCAATCAATACAACTTTCAACAACGGATCTCTTGGCTCTCGCATCGATGAAGAACGCAGCGAAATGCGATACGTAATGTGAATTGCAGAATTCAGTGAATCATCGAATCTTTGAACGCACCTTGCGCCCCTTGGTATTCCGAGGGGCACACCCGTTTGAGTGTCGTGAAATTCTCAAACCTTCTTGGTTTCTTGATCAAGATGGCTTTGGACTTTGGAGGCATTTGCTGGCTTTGCGAAAAGCCAGCTCCTCTTAAATGCATTAGTGGGGTCCCCTTTGCCGATCCCCAGGCGTGATAAGATGTTTCTACGTCTTGGGATTTGCTCTGTTCCTTGGGAACCTGCCTCTAACCGTCTCATGGAAGACATTGTTCGAGCTTGCTCGACCCACGAACCTTGACCTCAAATCGGGTGAGAC

>Russula persicina_MN704830

GTATGTGACCTGCGGAAGGTCATTATCGTATAACTGAGGCACAAGGGCTGTCGCTGACTTTTCGTCGTGCACGCCCAAAGTGCTCTCAATCATCCATCTCACCCCTATGTGCACCACCGCGTGGGTCCCCCTTTGGCTTGTCCTCAGGGGGGCTTGCGTTTTCATACAAACTCGATACAGTGTAGAATGTCTTTATTTTGCGGTAACACGCAATCAATACAACTTTCAACAACGGATCTCTTGGCTCTCGCATCGATGAAGAACGCAGCGAAATGCGATACGTAATGTGAATTGCAGAATTCAGTGAATCATCGAATCTTTGAACGCACCTTGCGCCCCTTGGCATTCCGAGGGGCACACCCGTTTGAGTGTCGTGAAATTCTCAAAACCCTTTCTTTTTAAGGATTTTGGACTTGAAGGCTTTTTTGCTGGCTTCCCATT

>Russula velenovskyi_MN704831

GTAGGTGACCTGCGGAGGATCATTATCGTATAACCGAGGTGCAAGGGCTGTTGCTGACCTTCAAAGGTCGTGCACGCCCAAGCACTCTCACACATCCATCTCTCACCCCCTTGTGCATCGCCGCGTGGGCCCCCCCTTTGCAGGAGGGCTTGCGTTTTCACATAAAACTTGATACAGTGTAGAATGTTTTCTTTTGCGGTCACACGCAATCAATACAACTTTCAACAACGGATCTCTTGGCTCTCGCATCGATGAAGAACGCAGCGAAATGCGATACGTAATGTGAATTGCAGAATTCAGTGAATCATCGAATCTTTGAACGCACCTTGCGCCCCTTGGCATTCCGAGGGGCACACCCGTTTGAGTGTCGTGAAATCATCAAAAATCTTTTTCTTTGAAAAGGATTTTTGGACTTGGAGGTTTCATGCTTGCTTTTGCCTTTGAAAGCGAGCTCCTCTGAAATAAATTAGTGGGGTCCGCTTTGCTGATCCTTAATGTGATAAGATGTTTCTACATTTTGGATTTAGCACTGTCCCTTGGACACCTGCTCCTAACTAGTTGTCCCTTGGACAATGATGGTGCTCCTGGTCACCGCCATCCACATTGGCGGGAGGCTGGACCCACAAAAGAAAAACCTTGACCTCAAA

>Russula xerampelina_MN704833

GACCTGCGGAGGATCATTATCGTACAACTGAGGTGCGAAGGCTGTCGCTGACCCTCAAAGGTCGTGCACGCCTGAGTGCTTTCACACAATCCATCTCACCTTTGTGCATCACCGCGTGGGTCCCCCTTTGCGGGGAGGGCTTGCGTTTTCACATAAAACTCGATACGGTGTAGAATGTTTCTTTTGCGGTCACACGCAATCAATACAACTTTCAACAACGGATCTCTTGGCTCTCGCATCGATGAAGAACGCAGCGAAATGCGATACGTAATGTGAATTGCAGAATTCAGTGAATCATCGAATCTTTGAACGCACCTTGCGCCCCTTGGCATTCCGAGGGGCACACCCGTTTGAGTGTCGTGAAATCTTCAAAAACCCTTTTCTCTTTGAACCATTGTGGTCGGGAAAGGGATTTTTGGACTTGGAGGTTTAATGCTCGCCCTTTCTTTCGAAAGCGAGCTCCTCTCAAATGAATTAGTGGGGTCCGCTTTGCTGATCCTTGACGTGATAAAATGTTTCTACGTTTTGGGTTTAGCACTGTCCCTTGGATGCCTGCTGCCAACCGTCTCATTGACAATGATGGTGCTCCGGTCACTGCCGTCTACATCGGCGGGAGGCTGGACCCACAAAAAGAAAACCTTGACCTCAAATCGGGTGAGACTACCCGCTGAACTTAAG

>Russula delica_MN704836

GTAGGTGACCTGCGGAAGGTCATTATCGTACAATGGGGGTACGACGGCTGTCGCTGACTTTAGTCGTGCACGCCCGAGTGCTCTCACATACAAATATCCATCTCACCCCTTTGTGCATCACCGCGTGGGTCCCCCTTCCTCGGAGGGGGTGCTCACGTTTTTAACATTAAACACCCATTCGAACGTAGTGTAGAATGTTCTTTGCGCGATCACGCGCGATCAATACAACTTTCAACAACGGATCTCTTGGCTCTCGCATCGATGAAGAACGCAGCGAAATGCGATACGTAATGTGAATTGCAGAATTCAGTGAATCATCGAATCTTTGAACGCACCTTGCGCCCCTTGGCATTCCGAGGGGCACACCCGTTTGAGTGTCGTGAACATCCTCAACCTGCTTGGTTGTATTGAACCAAGTAGGCTTGGAATTTGGAGGTTTTCTGCTGGCCTCCTCCGAAGCCAGCTCCTCTTAAATGTATTAGTGGGATCCGCTTTGCTAGATCCTCGACGTTGATAAGATGTTTCTACGTCTTGGGTTTCGCTCGGGAAAGGACCTGCTTCTAACCGTCCCATCGGGGACAACGTTCGAGCCGATCGCCCTTTACGGGGTGGGAAGCTTTTCGACCCATGAAACCTTGACCTCAAATCGGGTGAGACTACCCGCTGAACTTAAGCATATCAATAAGCGGAGGAAAAGAAACTAACAAGGATTCCCCTAGTAACTGCGAGTGAAGCGGGAAAAGCTCAATTTTAAAATCTGGTGGTCTTTTGGCCATCCGAGTTGTATTTAGAGAAG

>Russula sp 17 _MN737469

GGAAGGATCATTATCGTATAACTGAGGCACAAGGGCTGTCGCTGACTTTTCGTCGTGCACGCCCAAAGTGCTCTCAATCATCCATCTCACCCCTATGTGCACCACCGCGTGGGTCCCCCTTTGGCTTGTCCTCAGGGGGGCTTGCGTTTTCATACAAACTCGATACAGTGTAGAATGTCTTTATTTTGCGGTAACACGCAATCAATACAACTTTCAACAACGGATCTCTTGGCTCTCGCATCGATGAAGAACGCAGCGAAATGCGATACGTAATGTGAATTGCAGAATTCAGTGAATCATCGAATCTTTGAACGCACCTTGCGCCCCTTGGCATTCCGAGGGGCACACCCGTTTGAGTGTCGTGAAATTCTCAAAACCCTTTCTTTTTAAGGATTTTGGACTTGGAGGCTTTTTTGCTGGCTTCACATTGAAGCGAGCTCCTCTTAAATGGATTAGTGGGGTCTGCTTTGCCGATCCTTGACGTGATAAGATGCTTCTACGTCTCGGGCTTGGCATCGTGTACCTGCTTCTAACCGTCTCATCGACAATGATGGCGCTTCGGTCACCACCGTTTCATCGGTGGGAGGCTTGACCCACAAAAAAAACCTTGACCTCAAATCGGGTGAGACTACCCGCTGAACTTAAGCATATCAATAAGCGGAGGAAATCTCTAGAGGATCCCCGGGTACCGAGCTCGAATTCGTAATCATGGTCATAGCTGTTTCCTGTGTGAAATTGTTATCCGCTCACAATTCCACACAACATACGAGCCGGAAGCATAAAGTGTAAAGCCTGGGGTGCCTAATGAGTGAGCTAACTCACATTAATTGCGTTGCGCTCACTGCCCGCTTTCCAGTCGGGAAACCTGTCGTGCCAGCTGCATTAATGAATCGGCCAACGCGCGGGGAGAGGCGGTTTGCGTATTGGGCGCTCTTCCGCTTCCTCGCTCACTGACTCCGCTGCGCCTCGTCGTTCGGCTGCGGCGAGCGGTATCAGCCTCCCTCAAGGCGGTATACGTATCCACAGAATCAGGGGATAACGCAGAAAGAACATGTGAGCAAAGCAGCAAGCAGACCGTAAAAGCCGCGTGCTGGCGTTTTCCATAGCTCC

>Russula sp 18_MN737757

TAGGTGACCTGCGGAGGATCATTATCGTACAACCGGGGTGCAAGGGCTGTCGCTGACCCCTTCGAAGGTCGTGCACGTCCGAGCGCTCTCACCACAATCCATCTCACCCCTTTGTGCATCACCGCGTGGGTCCGCCTTTGCGGGAGGGCCCACGTTTTCACATAAAACTTGATACAGTCTAGAATGTTTATTTTTGCGGTAACACGCAATCAATACAACTTTCAACAACGGATCTCTTGGCTCTCGCATCGATGAAGAACGCAGCGAAATGCGATACGTAATGTGAATTGCAGAATTCAGTGAATCATCGAATCTTTGAACGCACCTTGCGCCCCTTGGCATTCCGAGGGGCACACCCGTTTGAGTGTCGTGAAATTCTCAAAATCCTTTTCTTTGACCGGAATAGGACTTTTGGACTTGGAGGTTCAATGCTTGCTTTCGCCTTTGAAAGCGAGCTCCTCTCAAATGAATTAGTGGGGTCCGCTTTGCTGATCCTTGACGTGATAAGATGTTTCTACGTTTTGGATTTGGCACTGTTTTCTCCGCTCCTAACTGTCTCATGGACAGCGATGGTGCTCCGGTCGCCGCCGTCTGTGGCGGGAGGCTGGACCCACAAAGAACCTTGACCTCAAATCGGGTGAGACTACCCGC

>Russula sp 19_MN737758

GTAGTGACCTGCGGAGGATCATTATCGTACAACCGGGGTGCAAGGGCTGTCGCTGACCCCTTCGAAGGTCGTGCACGTCCGAGCGCTCTCACCACAATCCATCTCACCCCTTTGTGCATCACCGCGTGGGTCCGCCTTTGCGGGAGGGCCCACGTTTTCACATAAAACTTGATACAGTCTAGAATGTTTATTTTTGCGGTAACACGCAATCAATACAACTTTCAACAACGGATCTCTTGGCTCTCGCATCGATGAAGAACGCAGCGAAATGCGATACGTAATGTGAATTGCAGAATTCAGTGAATCATCGAATCTTTGAACGCACCTTGTGCCCCTTGGCATTCCGAGGGGCACACCCGTTTGAGTGTCGTGAAATTCTCAAAATCCTTTTCTTTGACCGGAATAGGACTTTTGGACTTGGAGGTTCAATGCTTGCTTTCGCCTTTGAAAGCGAGCTCCTCTCAAATGAATTAGTGGGGTCCGCTTTGCTGATCCTTGACGTGATAAGATGTTTCTACGTTTTGGATTTGGCACTGTTTTCTCCGCTCCTAACTGTCTCATGGACAGCGATGGTGCTCCGGTCGCCGCCGTCTGTGGCGGGAGGCTGGACCCACAAAGAACCTTGACCTCAAATCGGGTGAGACTACCCGCTGAACTTAAGCATATCAATAAGCGGAGGAAAAGAAACTAACAAGGATTCCCCTAGTAACTGCGAGTGAAGCGGGAAAAGCTCAAATTTAAAATCTGGTGGTCTTTGGCCATCCGAGTTGTAATTAAAGAGCGTCTTCCGCCGCTGG

>Russula sp 20_MN737759

AGGTGACCTGCGGAGGTCATTATTGTACAACGGAGGTGCAAGGGCTGTCGCTGACCCTCAAAGGTCGTGCACGCCCGAGCGCGCTCTCACACAATCCATCTCACCCCTTTGTGCATCACCGCGTGGGTCCTCCCCCTTGCGGGAGGGCCTGCGTTTTCACATAAAACTCGATACAGTGTAGAATGTTCATTTTTGCGGTCACACGCAATCAATACAACTTTCAACAACGGATCTCTTGGCTCTCGCATCGATGAAGAACGCAGCGAAATGCGATACGTAATGTGAATTGCAGAATTCAGTGAATCATCGAATCTTTGAACGCACCTTGCGCCCCTTGGCATTCCGAGGGGCACACCCGTTTGAGTGTCGTGAAATTCTCAAAAACCCTTTCCTTTGATCGGATTTTTGGACTTGGAGGTTCAATGCTCGCCTTCAGCTTTTGAAAGCGAGCTCCTCTCAAATAAATTAGTGGGGTCCGCTTTGCTGGTCCTTGACGTGATAAGATGTTTCTACGTTTTCGACTTGGCACTGTCCCTTGGATCCTGCTTCTAACCTGTCCGTCTCACGGACAACGATGGTGCTCCGCTCACCGCCACTTACATCGGCGGGGAGGCTGGACCCACCAAAACGAACCTTGACCTCAAATCGGGTGAGACTACCCGCTGAACTTAAGCATATCAATAAGCGGAGGAAAAGAAACTAACAAGGATTCCCCTAGTAACTGCGAGTGAAGCGGGAAAAGCTCAAATTTAAAATCTGGTG

>Russula sp 21_MN737470

CGGAAGGTCATTATCGTACAACCGGGGTGCAAGGGCTGTCGCTGACCCCTTCGAAGGTCGTGCACGTCCGAGCGCTCTCACCACAATCCATCTCACCCCTTTGTGCATCACCGCGTGGGTCCGCCTTTGCGGGAGGGCCCACGTTTTCACATAAAACTTGATACAGTCTAGAATGTTTATTTTTGCGGTAACACGCAATCAATACAACTTTCAACAACGGATCTCTTGGCTCTCGCATCGATGAAGAACGCAGCGAAATGCGATACGTAATGTGAATTGCAGAATTCAGTGAATCATCGAATCTTTGAACGCACCTTGCGCCCCTTGGCATTCCGAGGGGCACACCCGTTTGAGTGTCGTGAAATTCTCAAAATCCTTTTCTTTGACCGGAATAGGACTTTTGGACTTGGAGGTTCAATGCTTGCTTTCGCCTTTGAAAGCGAGCTCCTCTCAAATGAATTAGTGGGGTCCGCTTTGCTGATCCTTGACGTGATAAGATGTTTCTACGTTTTGGATTTGGCACTGTTTTCTCCGCTCCTAACTGTCTCATGGACAGCGATGGTGCTCCGGTCGCCGCCGTCTGTGGCGGGAGGCTGGACCCACAAAGAACCTTGACCTCAAATCGGGTGAGACTACCCGCTGAACTTAAGCATATCAATAAGCGGAGGAAATCGTCGACCTGCAGGCATGCAAGCTTGGCACTGGCCGTCGTTTTACAACGTCGTGACTGGGAAAACCCTGGCGTTACCCAACTTAATCGCCTTGCAGCACATCCCCCTTTCGCCAGCTGGCGTAATAGCGAAGAGGCCCGCACCGATCGCCCTTCCCAACAGTTGCGCAGCCTGAATGGCGAATGGCGCCTGATGCGGTATTTTCTCC

>Russula sp 22_MN737471

ACGTAGGTGACCTGCGGAGGATCATTATCGTACAACCGAGGTGCGAGGGCTGTCGCTGACCTCCAAAGGTCGTGCACGCCCGAGCGCTCTCACACAATCCATCTCACACCCCCTTTGTGCATCACCGCGAGGGTCTTAGCTGTGGCTGTAACGGCCCCAAGGTGGGAATGTGAGGCGATGTTTTCCCCAACGGAGTCCCGTGCTCGTGCGCCGAGTTCAAGACTGTCCCCTCCAGAAAACTTTGGTGTCCATCGTTCCCCACGGCGTAGGCCGTGGTCGGATTTGGAGTAATCGGCCCTTGTCTAGCCAGGGAAGCAATTCCGCAGCTAGCAGGACTCACCGAGTTGGTCTTAGGAGATCAGGGCACGTCATCTGTCGCGCGGGTGCGCCGTGTCCCTCGTAGACCTTTATACCTCGCGTTTTCACACAAACCTCTGATACAGTGTAGAATGTTATTTTACCTTTTGCGGTCACACGCAATCAATACAACTTTCAACAACGGATCTCTTGGCTCTCGCATCGATGAAGAACGCAGCGAAATGCGATACGTAATGTGAATTGCAGAATTCAGTGAATCATCGAATCTTTGAACGCACCTTGCGCCCCTTGGCATTCCGAGGGGCACACCCGTTTGAGTGTCGTGAAATCATCAAAACCTTTTTCTCTTTGATCCCAAATTTATTTTGGTTGGGAAAAGGATTTTGGACTTGGAGGTTCCATGCTCGCTTTCAAAAGAAAGCGAGCTCCTCTCAAATGAATCAGTGGGGTCCGCTTTGCCGATCCTTGACGTGATAAGATGCTTCTACGTTTTGGATTTGGCACTGTCCCTTGGGCGCC

>Russula sp 23_MN737760

GTAGGTGACCTGCGGAGGATCATTATTGTATAACGGAGGTGCAAGGGCTGTCGCTGACCTTTAAAGGTCGTGCACGCCTAAGCCCTCTCACACAATCCATCTCACCCCCTTTTGTGCATCACCGCGTGGGTCCTCCCTTTGCCGGGAGGGCCTGCGTTTTTATATAAAACTTGACACGATGTAGAATGTTTTCTTTTTTGCAATCATATGCAATAAATACAACTTTCAACAACGGATCTCTTGGCTCTCGCATCGATGAAGAACGCAGCGAAATGCGATACGTAATGTGAATTGCAGAATTCAGTGAATCATCGAATCTTTGAACGCACCTTGCGCCCCTTGGCATTCCGAGGGGCACACCCGTTTGAGTGTCGTGAAATTCTCAAAAGCCCCCTTTTCTTGGAAAGGGAGTTTTGGACTTGGAGGTTCGATGCTTGCCTTTCACTTTTGAAAGCGAGCTCCTCTCAAATAAATTAGTGGGGTCCGCTTTGCTGATCCTTAACATGATAAGATGTTTCTATGTTTTGGATTTGGCACTGTCCCTTGGATGCCTGCTCCTAACTGTCTCACGGACAATGATGGTGTTTCGGTCGCTACCATTCATATCGGTGGAAGGCTGGACCCACGAAAATAAAACCTTTGACCTCAAATCGGGTGAGACTACCCGCTGA

>Russula sp 24_MN737761

GTAGGTGACCTGCGGAGGATCATTATCATACAACAGAGGTGCGAGGGCTGTCGCTGACCTTTTAAAGGTCGTGCACGCCCGAGTGCTCTCACACATCCATCTCACCCCTTTGTGCACAACCGCGTGAGTTCCCCTGGAAGGGGGGGCCCACGTTTTTTCACACAAACCTTAAAGCAGTGTAGAATGTATTTTCTTTTTGCGGTGATATGCGATCAATACAACTTTCAACAACGGATCTCTTGGCTCTCGCATCGATGAAGAACGTAGCGAAATGCGATATGTAATGTGAATTGCAGAATTCAGTGAATCATCGAATCTTTGAACGCACCTTGCGCCCCTTGGCATTCCGAGGGGCACACCCGTTTGAGTGTCGTGTAATCATCAAAACCTTTTCTTTGATCCTTTTGGTCGAGAAAGGGATTTTGGACTTGGAGGAATCAATGCTCGCTCTCACCTTTTGAAAGTGAGCTCCTCTTAAATAAATTAGTGGGGGTTTGCTTCGCCGATCCTTGACGTGATAAGTCGTTTCTACGTTTCGGATTCGGTTGGACACCCGCTTCTAACTGTCCTACGGACGATTGTGGTGTTCCGGTCACCTGGACCCATAAGAAAACCTTGACCTCAAATCGGGTGAGACTACCCGCTGAACTTAAGCA
