# Supplementary material for: The community composition variation of Russulaceae associated with the Quercus mongolica forest during the growing season at Wudalianchi City, China
Source: PeerJ. 2020 Feb 12;8:e8527. doi: 10.7717/peerj.8527 (PMC7023826; doi:10.7717/peerj.8527)
Supplement: Supplemental Information 6 [file peerj-08-8527-s006.doc]

**Table SRA accessions for root and soil samples**

| **Sample number** | **SRA accession** |
| --- | --- |
| Root 6 | **SRR10590047** |
| Root 7 | **SRR10590046** |
| Root 8 | SRR10590045 |
| **Root 9** | **SRR10590044** |
| Root 10 | **SRR10590043** |
| Soil6 | **SRR10590058** |
| Soil7 | **SRR10590057** |
| Soil8 | **SRR10590056** |
| Soil9 | **SRR10590055** |
| Soil10 | **SRR10590054** |
